# Effects of Lovastatin on Methane Production, Rumen Fermentation, and Microbial Composition In Vitro

**DOI:** 10.3390/microorganisms14102381

**Published:** 2026-10-18

**Authors:** Na Ren, Xianliu Wang, Jiaxin Qin, Zibin Zheng, Zhenyu Su, Chenxi Dong, Liwen He, Wei Zhang

**Affiliations:** State Key Laboratory of Animal Nutrition and Feeding, College of Animal Science and Technology, China Agricultural University, Beijing 100193, China

**Keywords:** lovastatin, methane production, rumen fermentation, microbiota, sheep

## Abstract

This study examined the effects of purified lovastatin (LV) obtained from *Monascus* fermentation on ruminal fermentation, methane production, and microbial communities during a 48-h in vitro incubation. A fattening-sheep diet containing concentrate and roughage at 60:40 was treated with 0, 50, 100, or 150 mg LV/kg dry matter (DM). Samples from the control and 100 mg/kg DM treatments were selected for bacterial and archaeal 16S rRNA amplicon sequencing. Compared with the control, supplementation with 100 mg LV/kg DM significantly decreased methane production (mL/g DM) and methane content (%) by 22.1% and 12.7%, respectively, while increasing in vitro DM degradation by 4.9%. Cumulative gas production and total VFA concentration did not differ significantly among treatments. The molar proportion of propionate was higher at 150 mg/kg DM, while MCP concentration was higher at 100 mg/kg DM. *Rikenellaceae RC9* gut group, *Eubacterium*, *Succinivibrionaceae UCG-001*, and *Anaerovibrio* were enriched in the LV treatment, whereas *Methanococcus* and *Methanosphaera* were enriched in the control. These results demonstrate the potential of lovastatin as a natural inhibitor of ruminal methanogenesis, with 100 mg/kg DM showing the greatest efficacy among the doses tested under the present in vitro conditions.

## 1. Introduction

Globally, livestock production contributes substantially to anthropogenic methane emissions. Approximately 37% of total methane emissions originate from livestock, while enteric fermentation by ruminants accounts for nearly 40% of greenhouse gas emissions from the agricultural sector [1,2]. Methane has a global warming potential approximately 25-fold greater than that of carbon dioxide and contributes as much as 22% of the greenhouse effect, thereby intensifying global climate change [3]. Moreover, ruminal methanogenesis causes an estimated loss of 2–12% of feed energy, thereby decreasing feed utilization efficiency [4]. Consequently, reducing enteric methane emissions is important for both the development of low-carbon livestock systems and improvements in the economic efficiency of ruminant production.

Current approaches to mitigating ruminal methane emissions mainly involve dietary intervention, genetic selection, and improvements in animal management [5]. Among these, modulating rumen microbial communities and fermentation patterns by supplementing diets with natural plant or fungal secondary metabolites has been demonstrated as a promising approach that simultaneously enhances feed utilization and suppresses methane production [6,7,8,9,10]. Lovastatin, a natural secondary metabolite produced by fungi such as *Monascus purpureus*, has garnered significant attention due to its inhibitory activity against 3-hydroxy-3-methylglutaryl-coenzyme A reductase (HMG-CoA reductase, HMGR) [11]. Previous in vitro studies indicate that lovastatin can restrict the growth and activity of *Methanobrevibacter*, the dominant methanogenic archaeal genus in the rumen of ruminants, which constitutes approximately 70–90% of total rumen methanogens. For instance, Miller and Wolin [12] observed that the addition of 4 mg/mL of pure lovastatin effectively inhibited the growth of *Methanobrevibacter* strains and reduced methane production in co-culture systems without adversely affecting cellulolytic bacteria. Similarly, in the rumen simulation technique (Rusitec) continuous in vitro systems, supplementation with 10 g/kg DM lovastatin reduced methane formation by 42% [13]. Faseleh Jahromi et al. [14] also observed a 24% methane suppression effect in vitro using *Aspergillus terreus* fermentation products, with no negative effect on DM digestibility. However, existing research findings on lovastatin’s methanogenesis-reducing effects in ruminants remain inconsistent. Previous studies have used *Aspergillus terreus*-fermented straw, fermented rice, and red yeast rice as carriers of lovastatin, resulting in 13–34% lower methane production in sheep or goats and potential improvements in DM digestibility [15,16,17,18]. In contrast, supplementation at a lower dose (1.06 mg/kg body weight/day) did not significantly reduce methane production [6], whereas high-dose lovastatin may decrease feed intake [19]. Additionally, fermentation by-products containing lovastatin often contain various secondary metabolites synthesized by fungi during fermentation, suggesting that the methanogenesis-suppressing effect may not be solely attributable to lovastatin [20]. Collectively, these discrepancies highlight an unresolved question of whether purified lovastatin itself inhibits *Methanobrevibacter* and suppresses methanogenesis in the complex rumen environment, or whether the effects previously attributed to lovastatin are partly dependent on co-occurring metabolites in crude fungal fermentation carriers.

We hypothesized that lovastatin supplementation would reduce methane production by selectively suppressing methanogenic archaea and shifting fermentation toward propionate, thereby lowering the acetate-to-propionate ratio. Therefore, graded levels of lovastatin were evaluated in an in vitro rumen fermentation system for their effects on fermentation characteristics, methane production, and microbial community structure. The objectives were to evaluate the methane-mitigation potential of lovastatin and examine associated fermentation and microbial patterns, thereby providing theoretical foundations and data support for developing natural, safe, and efficient methane suppression strategies for ruminants.

## 2. Materials and Methods

### 2.1. Animals and Design

The procedures involving donor sheep were approved by the Institutional Animal Care and Use Committee of China Agricultural University (approval no. AW31015202-1-5). The in vitro rumen fermentation experiment was conducted in two independent incubation batches using rumen fluid collected on separate dates from different donor sheep. Each batch involved three Hu sheep destined for slaughter, with an average body weight of 50 ± 2 kg. The donor sheep were fed the basal diet for 7 d before slaughter, with concentrate and roughage accounting for 60% and 40% of dietary DM, respectively. Details of the basal diet are provided in Table 1. A single-factor experimental design was adopted within each batch, with four levels of lovastatin (LV) supplementation: 0, 50, 100, and 150 mg/kg substrate DM. The 0 mg/kg LV group served as the ethanol control (CO; receiving 2 μL ethanol without lovastatin), and an additional no-ethanol control (NEC; receiving neither ethanol nor lovastatin) was included to assess the potential effects of the ethanol carrier used to solubilize lovastatin. Each treatment consisted of six fermentation replicates, and six blank tubes containing buffered rumen inoculum without substrate were included for background correction in each batch. Both batches followed the same experimental procedures and analytical methods, with fermentation tubes serving as within-batch experimental replicates.

### 2.2. Experimental Materials

Lovastatin (≥99% purity), purified from a *Monascus* sp. fermentation product, was obtained from Shanghai Yuanye Biotechnology Co., Ltd. (Shanghai, China) and supplied as a white crystalline powder. The lovastatin was supplied and introduced in its closed lactone form, which represents the stable, dominant species in crystalline powder at neutral pH. Before incubation, the required amount of lovastatin was dissolved in 2 μL of anhydrous ethanol and distributed over the substrate at 50, 100, or 150 mg/kg DM before inoculation. The ethanol was allowed to evaporate at room temperature before the buffered rumen inoculum was added. The CO treatment received 2 μL of anhydrous ethanol without lovastatin and was subjected to the same evaporation procedure to account for any potential solvent-handling effect.

### 2.3. Rumen Fermentation In Vitro

The fermentation procedure was performed with reference to the gas-production technique by Menke et al. [21]. Rumen fluid was taken separately from 3 donor sheep before feeding on the day of slaughter. After passage through 4 layers of gauze, the samples were pooled and mixed with two volumes of artificial saliva, yielding the buffered inoculum. During preparation, the inoculum was maintained at 39 °C under continuous stirring and continuously flushed with CO_2_ to maintain anaerobic conditions.

To prepare 1 L of artificial saliva, 400 mL of distilled water was combined with trace element A (0.1 mL), buffer B (200 mL), concentrated element C (200 mL), resazurin D [1 mL, 0.1% (*w*/*v*)], and reducing agent (40 mL). Distilled water was added to bring the final volume to 1 L. The inoculum was continuously flushed with CO_2_ until the redox indicator changed from red to colorless. The detailed makeup of Solutions A-D is given in Table 2.

After drying for 48 h at 65 °C, the substrate was milled to pass a 1.0-mm sieve. A 0.2000-g DM portion was then introduced into each prewarmed 100-mL graduated syringe. For each treatment, the lovastatin ethanol solution or 2 μL of anhydrous ethanol for the CO treatment was applied to the substrate. After the ethanol had evaporated at room temperature, each tube received 30 mL of buffered rumen inoculum. The headspace was then evacuated, the tubes sealed, and they were incubated immediately at 39 °C for 48 h in a constant-temperature incubator (DHP-9162, Shanghai, China). Gas accumulated in the syringes during incubation. When the syringe reading exceeded 60 mL, the accumulated gas was transferred to a gas bag for collection.

A separate 75-mL rumen-fluid system was set up to determine the in vitro DM degradation rate. A pre-weighed 0.5000 g portion of the basal diet was inserted into a fermentation flask. The amount of LV was adjusted according to the substrate DM, and the same solvent-handling procedure as described above was applied. After the ethanol had evaporated, 75 mL of buffered rumen inoculum was added to each flask. Following 48 h of incubation, the entire fermentation contents were filtered through a pre-weighed nylon filter (pore size 40 μm); the retained residues were washed with distilled water and dried at 65 °C to constant weight.

### 2.4. Gas Production and Methane Production

Gas production was measured directly by reading the graduations of the 100-mL syringes at 3, 6, 12, 24, 36, and 48 h of incubation. Cumulative gas production (GP) was corrected against the mean gas production of substrate-free blanks and expressed as mL/g DM. Methane concentration in the collected fermentation gas at 48 h was quantified according to Wu et al. [22].

Cumulative GP (mL/g DM) at each time point was calculated as follows:

Cumulative GP = (GP of sample tube − average GP of blank tubes)/DM.

The in vitro gas production curves were fitted using the NLIN procedure of SAS 9.4 (SAS Institute Inc., Cary, NC, USA) according to the following kinetic model:

GP = B(1 − e^(−ct)),

where B (mL/g DM) is the asymptotic gas production and c (h^−1^) is the rate of gas production per hour.

Methane production (mL/g DM) was calculated as follows:

Methane production (mL/g DM) = (GP of sample tube × methane concentration of sample tube − average GP of blank tubes × methane concentration of blank tubes)/DM.

### 2.5. Rumen Fermentation Parameters

Fermentation was terminated after 48 h by rapid cooling in an ice bath. The pH of the culture fluid was determined immediately after incubation. Volatile fatty acids were quantified by gas chromatography according to Wang et al. [23]. NH_3_-N and MCP were determined by the sodium nitroprusside–hypochlorite and Coomassie Brilliant Blue G-250 assays, respectively [24], with spectrophotometric detection.

### 2.6. Microbiome DNA Extraction and Amplicon Sequencing

To investigate the potential role of rumen microbial communities in lovastatin-mediated methane mitigation, bacterial and archaeal community analyses were conducted using fermentation samples from the control (CO) and 100 mg/kg DM lovastatin (LV) groups of the second independent incubation batch. Genomic DNA was isolated from 1 mL of fermentation fluid with a commercial DNA extraction kit (Omega Bio-Tek, Inc., Norcross, GA, USA). Bacterial community profiling covered the 16S rRNA V3–V4 region with 341F (5′-CCTAYGGGRBGCASCAG-3′) and 806R (5′-GGACTACNNGGGTATCTAAT-3′), whereas the archaeal V4–V5 region was examined with Arch519F (5′-CAGCCGCCGCGGTAA-3′) and Arch915R (5′-GTGCTCCCCCGCCAATTCCT-3′). Triplicate amplification products from each sample were combined before purification and quantification. Sequencing libraries were subsequently constructed and analyzed in paired-end mode on a DNBSEQ-G99 platform (MGI Tech Co., Ltd., Shenzhen, China) by Shanghai Biozeron Biotechnology Co., Ltd., Shanghai, China.

Sequence processing involved quality control with fastp (v0.20.0), paired-read assembly with FLASH (v1.2.7), chimera removal with USEARCH, and ASV inference through DADA2 (v1.16). Bacterial classification referred to SILVA release 138.2 with an 80% confidence threshold, whereas archaeal classification relied on the Rumen and Intestinal Methanogen Database (RIM-DB). Within-sample richness and diversity were summarized by ACE, Chao1, Shannon, and Simpson indices, which were calculated in R (version 4.4.2) using the vegan package. Between-group community variation was visualized via principal coordinate analysis (PCoA) based on Bray–Curtis dissimilarities, and statistical significance of group separation was tested by permutational multivariate analysis of variance (PERMANOVA) with 999 permutations. Both distance calculation and PERMANOVA were performed using the vegan package in R. Differentially abundant taxa between CO and LV groups were identified by linear discriminant analysis effect size (LEfSe) algorithm, implemented in R with the lefser package, using an LDA score above 3.0 and *p* < 0.05 as the screening thresholds.

### 2.7. Statistical Analysis

Statistical analyses of fermentation data were conducted using SAS 9.4. The Shapiro–Wilk and Levene tests were applied to verify normality and homogeneity of variance, respectively. Student’s t-test was used for pairwise comparison between the no-ethanol control (NEC) and ethanol control (CO). For the graded lovastatin treatments (0, 50, 100, and 150 mg/kg DM), gas production, methane traits, fermentation characteristics, MCP, and DM degradation were subjected to one-way ANOVA. Tukey’s HSD test was used for all pairwise comparisons among treatment groups. Linear and quadratic orthogonal polynomial contrasts were also performed to assess dose–response trends. Additionally, two-way ANOVA was performed to evaluate the effects of incubation batch, lovastatin treatment, and their interaction on gas production, methane production, and rumen fermentation parameters.

Amplicon data between CO and LV groups were analysed with the Wilcoxon rank-sum test, with Benjamini–Hochberg correction for multiple testing. Spearman rank-order correlation coefficients were calculated to assess associations between differential taxa and fermentation traits. PERMDISP was performed to test multivariate dispersion homogeneity between CO and LV. Statistical significance was set at *p* < 0.05.

## 3. Results

### 3.1. Gas Production, Methane Production and Kinetic Parameters from In Vitro Rumen Fermentation

The two independent incubation batches showed similar methane-mitigation responses to lovastatin supplementation, with the greatest reduction in methane production observed in the 100 LV group in both batches. Two-way ANOVA revealed no significant batch effect (*p* = 0.687) or batch × treatment interaction (*p* = 0.468) for methane production. Other measured parameters generally exhibited similar response patterns between the two batches. Results from the first batch are presented in Supplementary Tables S3 and S4, whereas those from the second batch are presented below.

As shown in Table 3, lovastatin supplementation did not significantly change cumulative gas production at any measured time point, asymptotic gas production, or the gas production rate (*p* > 0.05). Methane content decreased linearly (*p* = 0.005) and quadratically (*p* = 0.015) across lovastatin treatments (*p* = 0.003 for treatment effect), and was lower (*p* < 0.05) for the 100LV group than for the control, with a reduction of 12.7%. Methane production also decreased linearly (*p* = 0.045) and quadratically (*p* = 0.030) with increasing dosage (*p* = 0.012 for treatment effect), with the 100LV group showing a 22.1% decrease relative to the control (*p* < 0.05).

### 3.2. In Vitro Rumen Fermentation Parameters and DM Degradation Rate

As presented in Table 4, the in vitro DM degradation rate of the basal diet increased linearly (*p* = 0.021) and quadratically (*p* = 0.027) with increasing lovastatin levels, and was higher (*p* = 0.028) for the 100 LV group than for the control, with an increase of 4.9%. Lovastatin supplementation had no significant effect on pH, total VFA and individual VFA concentrations, the acetate-to-propionate ratio, or NH_3_-N concentration of rumen fluid (*p* > 0.05). The proportion of propionate increased linearly (*p* < 0.001) with lovastatin supply and was higher (*p* < 0.001) in the 150 LV group than in the control. MCP concentration was significantly increased linearly (*p* = 0.009) by lovastatin treatment; the 100 LV group showed 13.3% higher MCP concentration than the control (*p* < 0.001).

No significant differences were detected between NEC and CO across all measured indices, including gas production, methane production, kinetic parameters, DM degradation rate, VFA concentration, MCP, and NH_3_-N concentration (*p* > 0.05; Supplementary Tables S1 and S2).

### 3.3. Microbial Diversity and Composition

**Figure 1:**
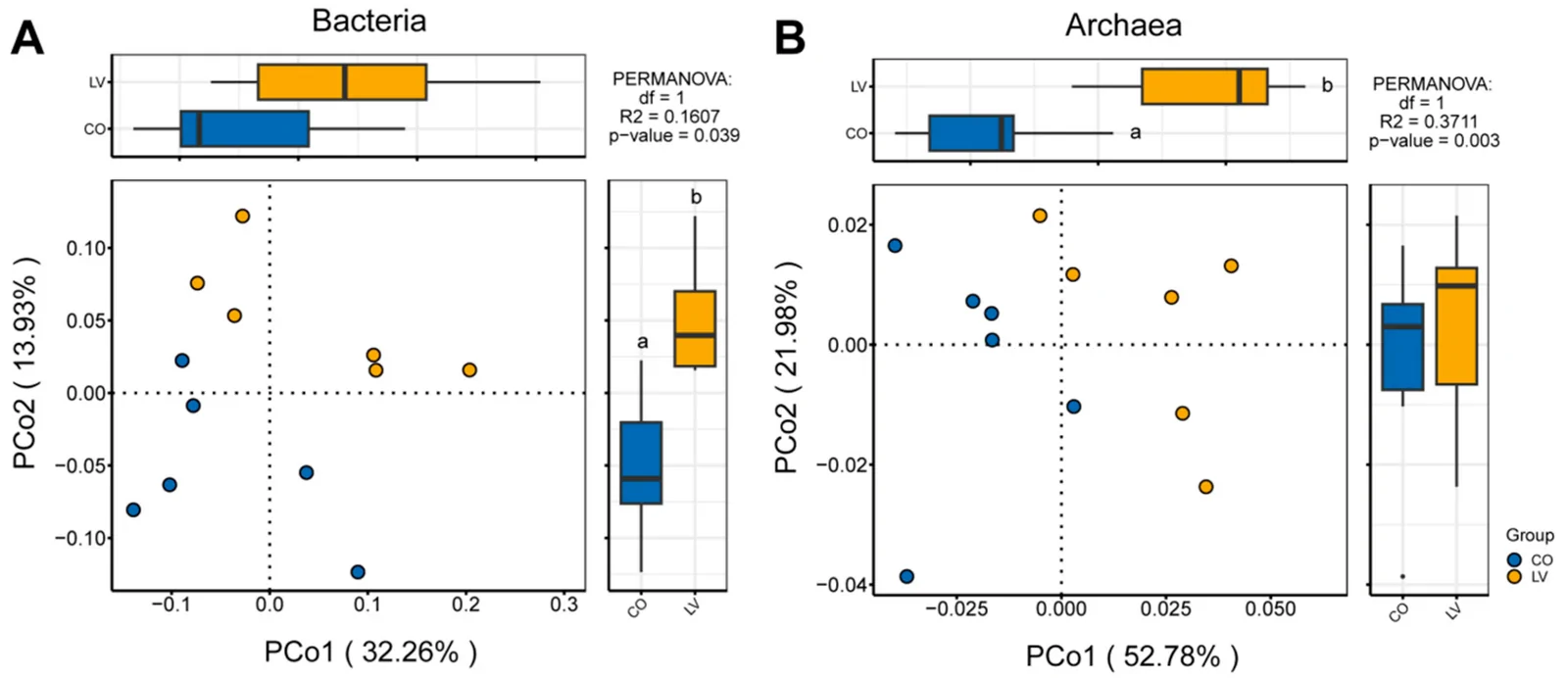
Bray–Curtis-based principal coordinate analysis showing separation of (**A**) rumen bacterial and (**B**) archaeal communities between the control (CO) and 100 mg/kg lovastatin (LV) groups. Group differences were assessed by PERMANOVA with 999 permutations. Different superscript letters indicate significant differences (*p* < 0.05).

Amplicon sequencing targeting the V3–V4 and V4–V5 regions of bacterial and archaeal 16S rRNA genes was carried out on 12 samples using primer pairs 341F/806R and Arch519F/Arch915R, respectively. Following rigorous quality control, including low-quality sequence filtering, paired-end sequence merging, and chimera removal, 422,355 bacterial and 431,622 archaeal sequences of high quality were obtained. All high-quality sequences were subsequently denoised using DADA2, yielding 1494 bacterial and 743 archaeal ASVs. Lovastatin supplementation did not significantly affect bacterial or archaeal richness (ACE, Chao1) or diversity (Shannon, Simpson) (Table 5). Bray–Curtis principal coordinate analysis indicated a distinct divergence in community structure between CO and LV groups for both bacteria (PERMANOVA: R^2^ = 0.161, *p* = 0.039) and archaea (PERMANOVA: R^2^ = 0.371, *p* = 0.003; Figure 1A,B), and PERMDISP testing confirmed homogeneous multivariate dispersion between groups (*p* > 0.05).

**Figure 2:**
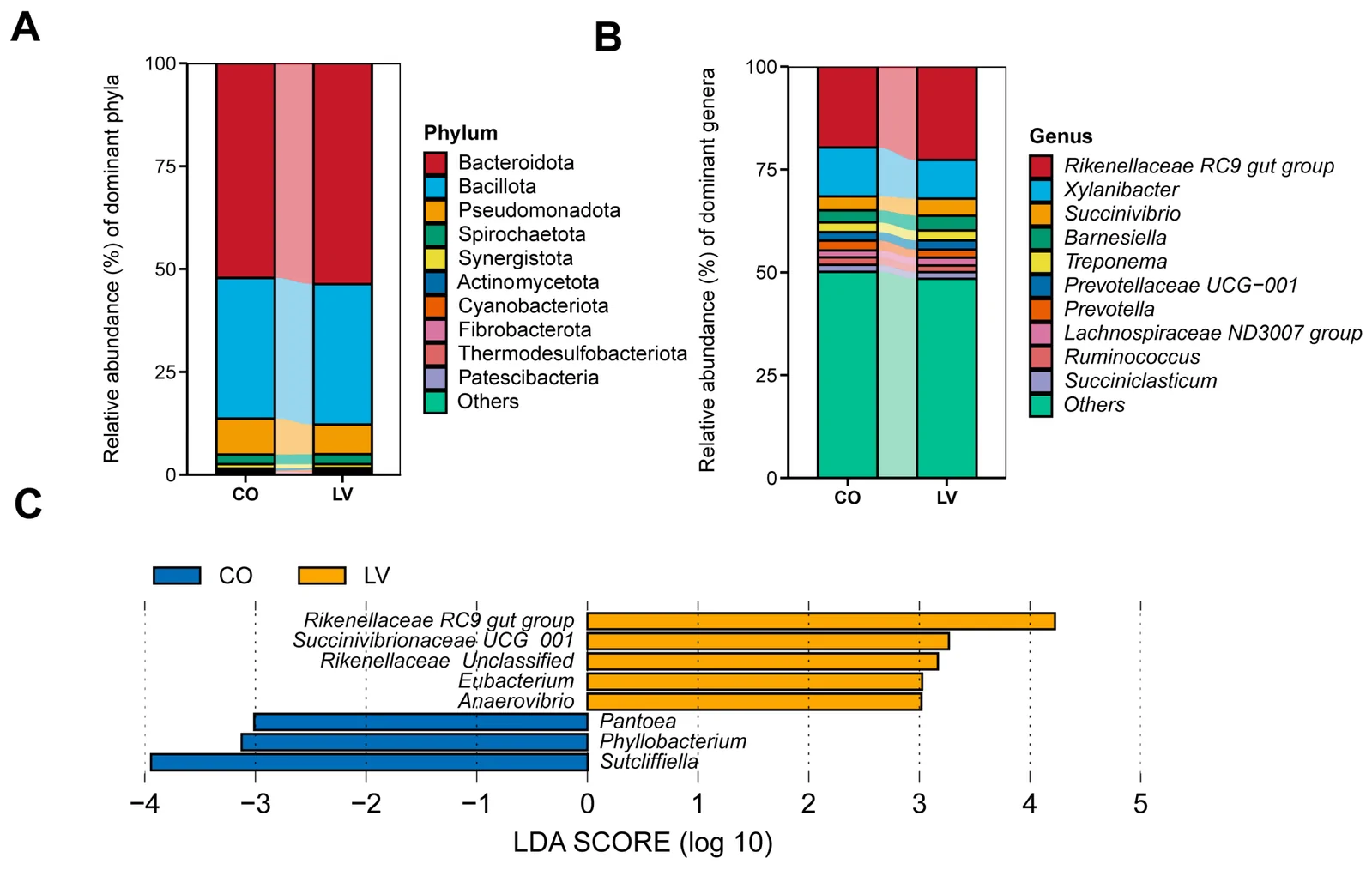
Bacterial community composition in the control (CO) and 100 mg/kg lovastatin (LV) groups based on 16S rRNA sequencing: (**A**) Phylum-level relative abundance; (**B**) Genus-level relative abundance; (**C**) Genus-level differential taxa identified by LEfSe analysis (LDA score > 3.0).

At the level of phylum, 16 bacterial phyla were detected, with Bacteroidota (52.91 ± 1.02%), Bacillota (34.17 ± 0.96%), and Pseudomonadota (7.99 ± 0.62%) being the predominant phyla (Figure 2A). At the level of genus, 20 bacterial genera exhibited mean relative abundances above 1%. The bacterial community was dominated by *Rikenellaceae RC9 gut group* (21.20 ± 0.77%) and *Xylanibacter* (10.63 ± 1.08%), followed by *Succinivibrio* (3.77 ± 0.21%), *Barnesiella* (3.25 ± 0.20%), *Treponema* (2.38 ± 0.27%), and *Prevotellaceae UCG-001* (2.17 ± 0.21%; Figure 2B). LEfSe analysis showed that the LV group was dominated by *Rikenellaceae RC9 gut group*, *Succinivibrionaceae UCG-001*, unclassified *Rikenellaceae*, *Eubacterium*, and *Anaerovibrio*, whereas *Pantoea*, *Phyllobacterium*, and *Sutcliffiella* were the dominant taxa in the CO group (LDA score > 3.0, *p* < 0.05; Figure 2C).

**Figure 3:**
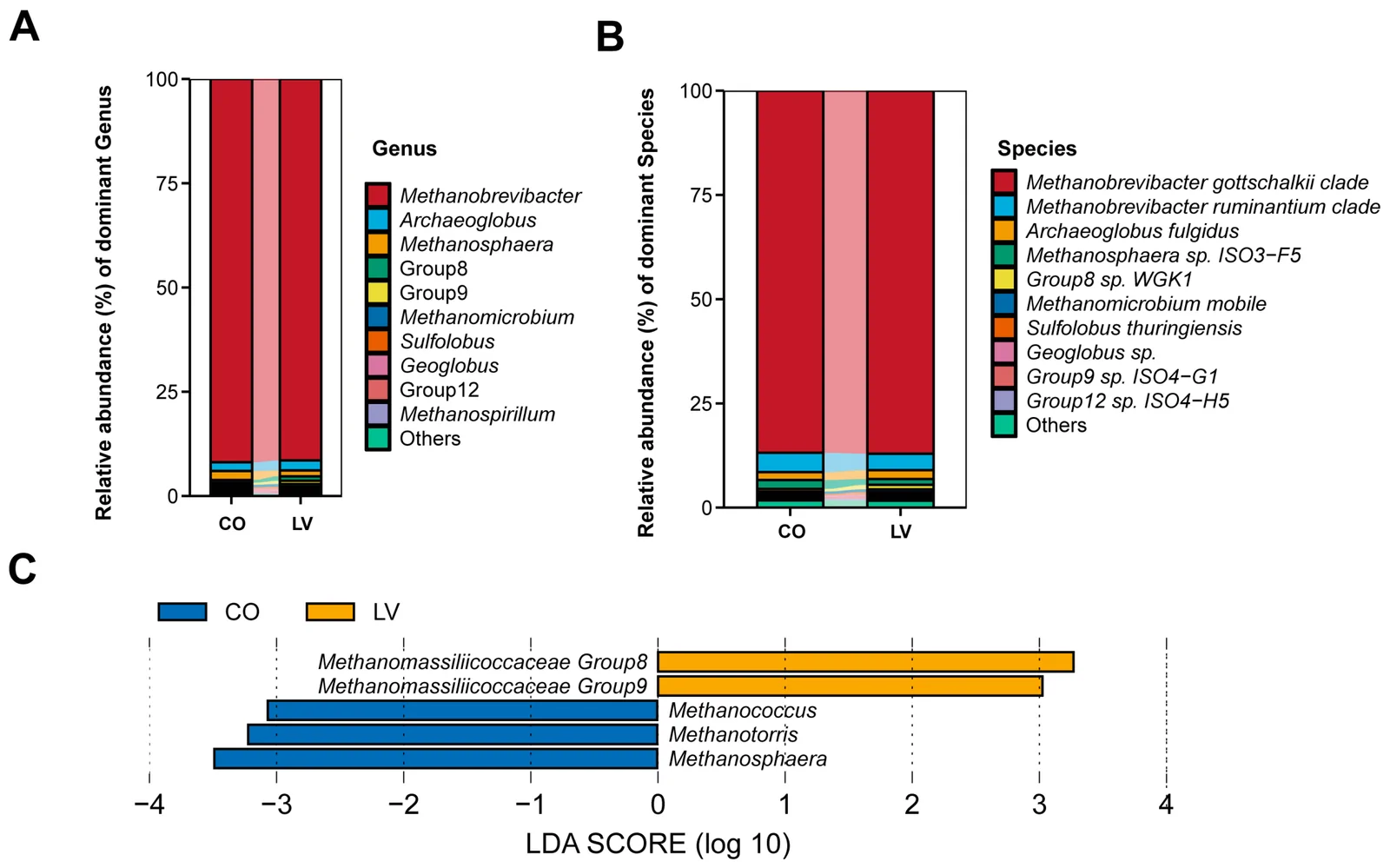
Archaeal community composition in the control (CO) and 100 mg/kg lovastatin (LV) groups based on 16S rRNA sequencing: (**A**,**B**) Genus- and species-level relative abundances; (**C**) LEfSe results for Genus-level differential taxa (LDA score > 3.0).

Based on RIM-DB taxonomic annotation, the archaeal community at the genus level was dominated by *Methanobrevibacter* (91.69 ± 0.69%), followed by *Archaeoglobus* (2.26 ± 0.27%), *Methanosphaera* (1.79 ± 0.13%), *Methanomassiliicoccaceae Group8* (0.85 ± 0.08%), *Methanomassiliicoccaceae Group9* (0.65 ± 0.05%), and *Methanomicrobium* (0.62 ± 0.18%; Figure 3A). At the species level, the predominant archaeal taxa were the *Methanobrevibacter gottschalkii* clade (86.96 ± 0.81%) and the *Methanobrevibacter ruminantium* clade (4.29 ± 0.20%), followed by *Archaeoglobus fulgidus* (2.02 ± 0.22%), *Methanosphaera* sp. *ISO3-F5* (1.75 ± 0.13%), *Group8* sp. *WGK1* (0.85 ± 0.08%), and *Methanomicrobium mobile* (0.62 ± 0.18%; Figure 3B). LEfSe analysis showed significant enrichment of *Methanomassiliicoccaceae Group8* and *Group9* in the LV group, and of *Methanococcus*, *Methanotorris*, and *Methanosphaera* in the CO group (LDA score > 3.0, *p* < 0.05; Figure 3C).

**Figure 4:**
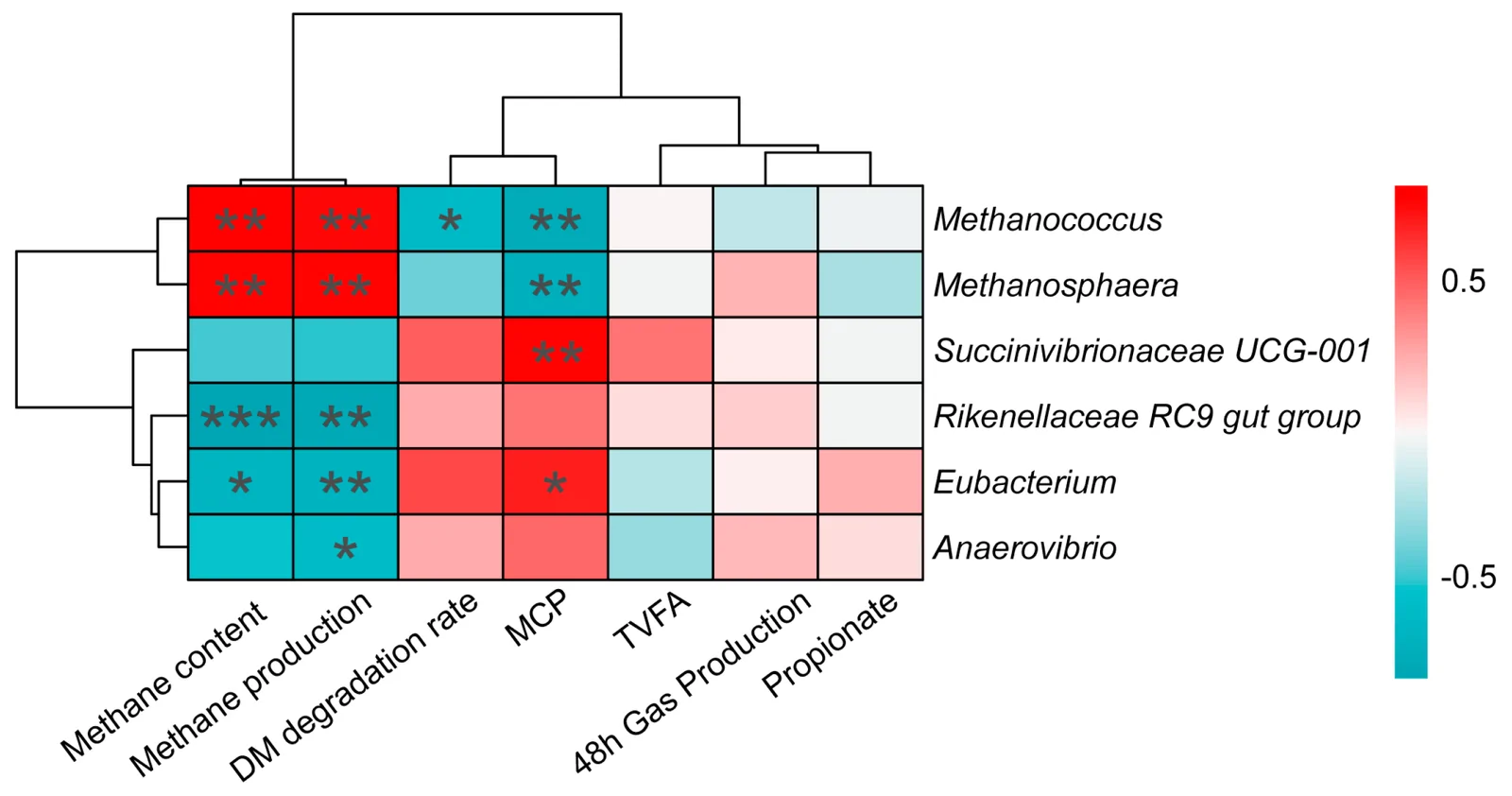
Correlation heatmap of rumen fermentation and gas production parameters with differentially abundant microbial taxa. Coefficients (positive and negative) are shown on the right; statistical significance is denoted by asterisks: * *p* < 0.05, ** *p* < 0.01, and *** *p* < 0.001. TVFA = total VFA.

Spearman analysis (Figure 4) indicated that methane production correlated negatively with the relative abundances of *Rikenellaceae RC9 gut group*, *Eubacterium*, and *Anaerovibrio*, while methane content correlated negatively with the relative abundances of *Rikenellaceae RC9 gut group* and *Eubacterium* (*p* < 0.05). MCP concentration was positively associated with the relative abundances of *Eubacterium* and *Succinivibrionaceae UCG-001* (*p* < 0.05). For archaea, the relative abundance of *Methanosphaera* was positively linked to methane content and production but negatively to MCP concentration; similarly, the relative abundance of *Methanococcus* also positively correlated with methane parameters, but its correlations with DM degradation rate and MCP were negative (*p* < 0.05; Figure 4).

## 4. Discussion

Methane production is influenced by multiple factors, including the composition of feed ingredients, the type of rumen fermentation, and the microbial community [5]. In this study, pure lovastatin supplementation significantly reduced methane content by 12.7% and methane production by 22.1%, in line with the lower abundances of *Methanosphaera* and *Methanococcus* in the LV group, both of which were positively correlated with methane production. This finding is consistent with the proposed anti-methanogenic mechanism of lovastatin, whereby inhibition of HMGR may interfere with isoprenoid membrane-lipid biosynthesis in methanogenic archaea [10]. However, a consensus has not yet been reached regarding the effects of pure lovastatin on methane reduction in vitro and in vivo. Although trials using fermentation by-products as lovastatin carriers reported methane reductions [14,18,19], pure lovastatin has not shown consistent effects [15,25]. This discrepancy may be attributed to the adsorption of lovastatin onto solid media in rumen inocula, thereby reducing its bioavailability [10]. Another possible explanation is that crude fungal fermentation products contain secondary metabolites in addition to lovastatin, which may act synergistically to enhance methane mitigation [26,27]. The approximately 22.1% reduction in methane production (a 12.7% decrease in the proportion of methane) observed with the purified lovastatin (purity ≥ 99%) used in the present study is consistent with the previously reported 23.7% reduction in methane production (a 10% decrease in the proportion of methane) by an *Aspergillus terreus* fermentation product [14], confirming that pure lovastatin exhibits an effective capacity for methane mitigation. Furthermore, an in vitro study conducted by Ábrego-García et al. [28] confirmed that pure lovastatin at 80 and 160 mg/L decreased in vitro ruminal methane emissions, with a stronger inhibitory effect in sheep diets than in goat or dairy cow diets. Notably, the lovastatin level used in that study was 100 times the in vivo supplementation level, which may limit its practical application in animal feeding. The present study employed purified lovastatin derived from *Monascus* fermentation at doses closer to those used in vivo, and further confirmed that pure lovastatin is capable of mitigating methane emissions. However, the response was not dose-proportional: the greatest reduction occurred at 100 mg/kg DM, whereas increasing the dose to 150 mg/kg DM provided no additional mitigation benefit.

Importantly, lovastatin supplementation did not significantly affect cumulative ruminal gas production at any incubation time point, consistent with Morgavi et al. [15]. As a key indicator reflecting ruminal microbial substrate fermentation [29], the maintenance of gas production suggests that lovastatin reduced methane formation without broadly suppressing substrate fermentation. Under high-roughage dietary conditions, Ábrego-García et al. [28] also found that 72-h in vitro gas production remained unchanged with dietary lovastatin supplementation, potentially due to a concurrent increase in hydrogen production alongside reduced methane production. However, another study reported that a lovastatin-containing fermented rice straw extract significantly reduced cumulative gas production after 12 h, primarily because of decreased methane production, together with a reduction in hydrogen production [14]. These findings suggest that the inconsistent effects of lovastatin on gas production may be related to dietary composition.

The regulation of rumen fermentation parameters by lovastatin further elucidates its metabolic mechanism for methane reduction. Rumen pH, negatively correlated with total VFA concentration [30], serves as a crucial indicator of rumen environment. pH below 6.2 inhibits fibre degradation and microbial metabolism [31]. No significant change in ruminal pH was observed with lovastatin, alongside stable total VFA levels, implying that microbial growth and feed nutrient digestion were not adversely affected. Lovastatin also increased dietary DM degradation rates, corroborating Mohd Azlan et al. [17], who observed similar improvements in goat DM digestibility. The improvement in DM degradation rate may be associated with the selective regulation of rumen microbial communities by lovastatin. Lovastatin reduced the relative abundance of taxa such as *Sutcliffiella* and increased those of *Rikenellaceae RC9 gut group*, *Succinivibrionaceae UCG-001*, and *Eubacterium*, which have been linked to the decomposition and utilization of both structural and non-structural carbohydrates in previous studies [32].

The composition characteristics of ruminal VFA reflect shifts in fermentation patterns. In this study, the 150 mg/kg DM treatment significantly increased the molar percentage of propionic acid, suggesting that lovastatin at a higher dose may influence hydrogen partitioning through changes in fermentation end products [33]. This aligns with findings showing a shift towards propionic acid fermentation in rumen fermentation patterns following lovastatin supplementation in goat and cattle diets [18,19]. This shift in fermentation pattern not only facilitates methane reduction but also enhances feed energy utilisation efficiency. This is because propionic acid possesses a higher energy value (2.51 MJ/mol) than acetic acid (1.78 MJ/mol) and exhibits greater efficiency in conversion to glucose, positively impacting growth performance in finishing sheep [34]. Additionally, the significantly elevated MCP content in the 100 mg/kg groups, along with unchanged NH_3_-N levels, suggests that the effects of lovastatin could enhance microbial protein synthesis and improve nitrogen utilization. Dietary crude protein is microbially degraded to produce ammonia, which can be assimilated by rumen microorganisms for MCP synthesis [35]. Current research has not addressed the impact of lovastatin supplementation on microbial protein, but studies in sheep and goats found no adverse effects on nitrogen metabolism [6,18]. Consistent with these findings, Ábrego-García et al. [28] attributed the significant reduction in ruminal NH_3_-N concentration observed in vitro following lovastatin supplementation to enhanced microbial nitrogen capture.

This study found no significant effect of lovastatin on α-diversity of rumen bacteria and methanogenic archaea, which aligns with previous research findings that lovastatin does not alter the α-diversity of the rumen microbiota when methane production is inhibited [27]. However, the bacterial and archaeal communities in the control and lovastatin groups were significantly separated by Bray–Curtis PCoA, indicating that lovastatin altered rumen microbial composition. Lovastatin significantly increased the abundances of cellulolytic genera such as *Rikenellaceae RC9 gut group* and *Eubacterium*, consistent with the improved DM degradation rate [32,36]. The dominant Hu sheep rumen genus *Rikenellaceae RC9 gut group* contributes to fiber fermentation and VFA production [37], whereas *Eubacterium*, a key cellulolytic bacterium, decomposes structural carbohydrates and correlates positively with NDF digestibility [38]. The enhancement of cellulolytic bacteria by lovastatin-rich fermented rice straw extracts, as reported by Faseleh et al. [14], lends support to our current results. A notable finding of the present study was the enrichment of *Succinivibrionaceae UCG-001*, a genus involved in hydrogen utilization in the rumen and carbohydrate fermentation to succinate [39], a key precursor for propionate synthesis [40]. In the 100 mg/kg DM group, propionate molar proportion did not rise significantly, yet *Succinivibrionaceae UCG-001* enrichment may indicate a microbial shift toward succinate-producing pathways. Increased *Succinivibrionaceae* abundance may enhance competition with hydrogenotrophic methanogens for hydrogen [41], potentially contributing to lower methane production. From a thermodynamic perspective, the shift to succinate fermentation diverts reducing equivalents from Fdred-dependent H_2_ evolution to succinate synthesis, thereby reducing steady-stateH_2_ partial pressure in closed in vitro batch systems and preserving the thermodynamic favorability of high-ATP-yield pathways otherwise inhibited by H_2_ accumulation [42]. *Anaerovibrio* was enriched in the LV group and was negatively correlated with methane production. The observed enrichment of *Anaerovibrio* was closely associated with propionate production [43], as *Anaerovibrio* can ferment glycerol to produce propionate and succinate [44,45].

In the LV group, *Methanosphaera* and *Methanococcus* positively correlated with methane content and production, while their abundances were reduced, potentially contributing to the lower methane yield [46]. Wang et al. [16] reported similar reductions in *Methanosphaera* abundances with red yeast rice supplementation. These findings suggest that lovastatin may affect multiple methanogenic taxa rather than a single substrate-utilization pathway, potentially through interference with isoprenoid membrane-lipid biosynthesis, a conserved feature of methanogenic archaea [10,47]. Notably, *Methanomassiliicoccaceae Group8* and *Group9*, two H_2_-dependent methylotrophic methanogen lineages within the order Methanomassiliicoccales, showed higher relative abundances in the LV group despite the reduction in methane production [48]. Moreover, some Methanomassiliicoccales genera were actively transcribed at higher levels in low-methane-emitting ruminants, indicating that the enrichment of specific members of this order does not necessarily correspond to increased methane production [48].

In contrast to previous studies that reported a reduction in *Methanobrevibacter* abundance following lovastatin treatment [10], the relative abundance of this predominant archaeal genus, which accounted for more than 91% of the archaeal community, was not significantly altered in the present study. Combined with the differential responses of the aforementioned taxa, this demonstrates that although HMGR-dependent mevalonate metabolism is broadly involved in archaeal isoprenoid membrane-lipid biosynthesis, this shared biochemical target does not necessarily result in uniform suppression across all methanogenic lineages, and susceptibility to lovastatin varies across taxa. Similarly, Wang et al. [16] found that red yeast rice supplementation decreased the relative abundance of *Methanobrevibacter* but increased that of *Methanomicrobium*. The inconsistent response of *Methanobrevibacter* across studies may arise from cross-study variations in dietary concentrate-to-roughage ratio, incubation duration, and lovastatin dosage. Furthermore, partial adsorption of lovastatin by fibrous dietary substrates in the culture medium may reduce its actual bioavailability to the ruminal microbiota [10]. Previous studies have demonstrated that lovastatin reduces methane production by suppressing the gene expression of F420-dependent oxidoreductase in *Methanobrevibacter smithii*, which modulates methanogenic metabolism at the transcriptional level and further interferes with electron transfer and energy metabolism [49,50]. Given that 16S rRNA amplicon sequencing reflects only relative abundance rather than absolute abundance or metabolic activity, the specific mechanism by which lovastatin reduces methane—whether through inhibiting methanogen abundance, suppressing key enzyme gene expression, or a combination of both—remains to be further verified, and functional validation using approaches such as mcrA quantification and metatranscriptomic analysis is required to clarify its direct effects on methanogenesis. Additionally, although lovastatin was introduced in its stable lactone form, its dynamic chemical stability, adsorption kinetics, and in situ hydrolysis into the bioactive open hydroxyacid form were not tracked over the 48-h incubation; characterizing these metabolic transitions represents an important objective for future mechanistic studies.

From an applied perspective, the high extraction and purification costs of pure lovastatin represent a substantial economic hurdle for commercial ruminant production, precluding its routine use as a dietary additive for enteric abatement. Here, purified lovastatin served as a mechanistic proof-of-concept; a more commercially viable strategy involves solid-state fermentation (SSF) of low-quality roughages using lovastatin-producing, lignocellulose-degrading fungi such as *Monascus* spp. and *Aspergillus terreus* [14,15,27]. This bioconversion approach offers dual nutritional and environmental advantages: First, fungal lignocellulolytic enzymes disrupt the recalcitrant fiber matrix and reduce substrate fiber content, enhancing the ruminal degradability and feed efficiency of agricultural residues without impairing rumen digestive equilibrium [26,27]. Supporting this, fermented agricultural biomass enriched with fungal mycelia exhibited markedly higher ruminal digestibility than untreated rice straw [51]. Second, the fungal biomass accumulates lovastatin, converting low-value agricultural residues into cost-effective, in situ delivery vehicles that effectively suppress enteric methanogenesis while promoting environmentally sustainable ruminant production [14,18,19].

As an in vitro investigation, this experiment could not fully replicate the complex and dynamic in vivo ruminal environment, particularly regarding the epithelial absorption of fermentation end-products and the passage rate of digesta [30,52]. Furthermore, although the fermentation experiment was conducted in two independent incubation batches, microbial community analysis was performed only on samples from the second batch, which may limit the generalizability of the microbial findings. While these results demonstrate the anti-methanogenic potential of *Monascus*-derived purified lovastatin, further validation across additional independent incubation batches and in vivo feeding trials remains necessary to evaluate its effects on methane production, host nitrogen utilization efficiency, and production performance.

## 5. Conclusions

Supplementation with 100 mg/kg DM lovastatin reduced methane production by 22.1% while increasing DM degradation and MCP concentration under the present in vitro conditions. Microbial analysis showed that *Methanosphaera* and *Methanococcus* were enriched in the CO group, whereas *Rikenellaceae RC9 gut group*, *Eubacterium*, and *Succinivibrionaceae UCG-001* were enriched in the LV group. Overall, lovastatin demonstrated potential as a natural inhibitor of ruminal methanogenesis, with 100 mg/kg DM exhibiting the greatest anti-methanogenic efficacy among the doses evaluated under the present 48-h in vitro conditions.

## Figures and Tables

**Table 1:** Ingredient and nutrient contents of the basal diet on a DM basis.

Item	Content %
Corn	38.00
Rapeseed meal	5.00
Soybean meal	6.00
Distillers dried grains with solubles (DDGS)	3.60
Corn germ meal	3.18
Sodium bicarbonate	0.90
Calcium carbonate	1.20
Salt	0.60
Dicalcium phosphate	0.80
Corn stover silage	22.00
Dry distillers’ grains	18.00
Premix ^1^	0.72
Total	100
Nutritional composition	
OM	89.57
CP	14.78
NDF	38.69
ADF	22.72
Metabolisable energy ^2^, MJ/kg	9.59
Ca, %	1.08
P, %	0.58

^1^ Premix provided per kilogram of premix: Vitamin A 800,000 IU, Vitamin E 3000 mg, Vitamin D_3_ 300,000 IU, Zn 5000 mg, Cu 800 mg, Mn 4000 mg, Fe 4000 mg, I 70 mg, Se 20 mg, Co 40 mg. ^2^ Metabolisable energy is a calculated value, expressed in MJ/kg of dry matter. DM, dry matter; OM, organic matter; CP, crude protein; NDF, neutral detergent fiber; ADF, acid detergent fiber.

**Table 2:** Composition of artificial saliva stock solution.

Trace element (Solution A):		
	CoCl_2_·6H_2_O	1.0 g
	FeCl_3_·6H_2_O	8.0 g
	MnCl_2_·4H_2_O	10.0 g
	CaCl_2_·2H_2_O	13.2 g
	Distilled water to	100 mL
Buffer (Solution B):		
	NaHCO_3_	35.0 g
	NH_4_HCO_3_	4.0 g
	Distilled water to	1000 mL
Concentrated element (Solution C):		
	Na_2_HPO_4_	5.7 g
	KH_2_PO_4_	6.2 g
	MgSO_4_·7H_2_O	0.6 g
	Distilled water to	1000 mL
Resazurin (Solution D):		0.1% (*w*/*v*)
Reducing agent solution:		
	1N NaOH	4.0 mL
	Na_2_S·9H_2_O	625.0 mg
	Distilled water	95 mL

**Table 3:** Effects of Lovastatin on gas production, methane production and kinetic parameters in vitro.

Items	Group ^1^				SEM ^2^	*p*-Value		
	CO	50 LV	100 LV	150 LV		Treatment	Linear	Quadratic
Cumulative gas production (mL/g)								
3 h	36.06	42.66	35.07	35.76	2.20	0.605	0.673	0.521
6 h	75.62	88.67	71.50	73.19	3.15	0.201	0.367	0.359
12 h	158.14	163.63	143.68	152.28	4.23	0.432	0.327	0.857
24 h	195.19	202.84	193.40	202.48	4.29	0.835	0.758	0.938
36 h	211.53	216.92	207.70	218.20	4.70	0.874	0.808	0.800
48 h	216.69	221.58	213.16	222.89	4.54	0.889	0.813	0.805
B (mL/g)	224.19	226.89	223.17	234.14	9.42	0.842	0.556	0.635
c (h^−1^)	0.083	0.091	0.076	0.076	0.002	0.062	0.072	0.332
Methane content (%)	22.01 ^a^	20.92 ^ab^	19.22 ^b^	20.49 ^ab^	0.294	0.003	0.005	0.015
Methane production (mL/g)	49.99 ^a^	46.88 ^a^	38.92 ^b^	45.80 ^ab^	1.31	0.012	0.045	0.030

^1^ CO: control (without lovastatin); 50 LV, 100 LV, and 150 LV, lovastatin added at 50, 100, and 150 mg/kg DM, respectively; ^2^ SEM: standard error of the mean; B: asymptotic gas production per gram DM substrate; c: the rate of gas production per hour. Different superscript letters indicate significant differences (*p* < 0.05).

**Table 4:** Effects of Lovastatin on fermentation parameters and DM degradation rate in vitro.

Items	Group ^1^				SEM ^2^	*p*-Value		
	CO	50 LV	100 LV	150 LV		Treatment	Linear	Quadratic
DM degradation rate (%)	61.57 ^b^	63.73 ^ab^	64.60 ^a^	63.70 ^ab^	0.415	0.028	0.021	0.027
pH	6.90	6.91	6.92	6.91	0.007	0.930	0.758	0.571
TVFA concentration (mM)	143.11	142.17	144.26	143.04	1.076	0.934	0.856	0.954
Acetate (mM)	76.79	76.42	77.37	77.49	0.688	0.949	0.649	0.868
Propionate (mM)	27.27	27.06	27.70	27.70	0.230	0.723	0.382	0.823
Butyrate (mM)	21.44	21.08	21.38	20.67	0.119	0.077	0.049	0.439
Isobutyrate (mM)	3.18	3.18	3.22	3.13	0.019	0.416	0.490	0.213
Valerate (mM)	4.80	4.81	4.89	4.73	0.033	0.439	0.671	0.218
Isovalerate (mM)	9.62	9.61	9.71	9.33	0.069	0.229	0.201	0.183
mol/100 mol VFA								
Acetate	53.66	53.75	53.63	54.13	0.104	0.232	0.100	0.446
Propionate	19.06 ^b^	19.03 ^b^	19.20 ^ab^	19.36 ^a^	0.036	<0.001	<0.001	0.079
Butyrate	14.98	14.83	14.82	14.48	0.070	0.061	0.013	0.462
Isobutyrate	2.22	2.24	2.23	2.19	0.013	0.600	0.425	0.277
Valerate	3.35	3.39	3.39	3.31	0.016	0.294	0.399	0.095
Isovalerate	6.72	6.76	6.73	6.53	0.035	0.067	0.042	0.075
Acetate:Propionate	2.82	2.82	2.79	2.80	0.005	0.106	0.056	0.760
NH_3_-N (mg/dL)	30.49	30.47	31.24	30.60	0.212	0.606	0.574	0.487
MCP (mg/dL)	2.93 ^bc^	2.60 ^c^	3.32 ^a^	3.08 ^ab^	0.069	<0.001	0.009	0.626

^1^ CO: control (without lovastatin); 50 LV, 100 LV, and 150 LV: lovastatin added at 50, 100, and 150 mg/kg DM, respectively; ^2^ SEM: standard error of the mean. TVFA: total volatile fatty acid; NH_3_-N: ammonia nitrogen; MCP: Microbial Crude Protein. Different superscript letters indicate significant differences (*p* < 0.05).

**Table 5:** Bacterial and archaeal alpha diversity in vitro.

Items	Groups		SEM	*p*-Value
	CO	LV		
Bacteria				
ACE	564.55	582.86	7.63	0.272
Chao1	564.36	582.64	7.65	0.272
Shannon	5.55	5.57	0.016	0.574
Simpson	0.99	0.99	0	0.763
Good’s coverage (%)	99.86	99.84	0.02	0.412
Archaea				
ACE	192.09	186.10	11.5	0.806
Chao1	191.77	186.04	11.6	0.813
Shannon	2.75	2.77	0.142	0.957
Simpson	0.79	0.79	0.016	0.889
Good’s coverage (%)	99.92	99.91	0.01	0.554

Group1 CO: control (without lovastatin); LV, lovastatin added at 100 mg/kg DM; SEM2: standard error of the mean.

## Data Availability

The 16S rRNA gene sequencing data generated in this study have been deposited in the National Center for Biotechnology Information (NCBI) Sequence Read Archive (SRA) under BioProject accession number PRJNA1438302.

## Supplementary Materials

The following supporting information can be downloaded at: https://www.mdpi.com/article/10.3390/microorganisms14102381/s1, Table S1. Effects of the ethanol carrier on gas production, methane production, and kinetic parameters in vitro. Table S2. Effects of the ethanol carrier on fermentation parameters and DM degradation rate in vitro. Table S3. Effects of Lovastatin on gas production, methane production and kinetic parameters in the first in vitro fermentation batch. Table S4. Effects of Lovastatin on fermentation parameters and DM degradation rate in the first in vitro fermentation batch.

## Abbreviations

The following abbreviations are used in this manuscript:

ASV	Amplicon Sequence Variant
CO	Control
HMG-CoA	3-hydroxy-3-methylglutaryl-coenzyme A
LDA	Linear Discriminant Analysis
LV	Lovastatin
MCP	Microbial Crude Protein
NH_3_-N	Ammonia Nitrogen
PCoA	Principal Coordinate Analysis
SRA	Sequence Read Archive
