# Supplementary material for: Effects of Lovastatin on Methane Production, Rumen Fermentation, and Microbial Composition In Vitro
Source: Microorganisms. 2026 Oct 18;14(10):2381. doi: 10.3390/microorganisms14102381 (PMC13707627; doi:10.3390/microorganisms14102381)
Supplement: Supplementary file 1 [file microorganisms-14-02381-s001.zip › microorganisms-4621199-supplementary.pdf]

Additional Tables

Table S1. Effects of the ethanol carrier on gas production, methane production, and kinetic parameters *in vitro*

Items	NEC[1]	CO	SEM[2]	*P*-value
Cumulative gas production (mL/g)				
3 h	32.32	36.06	3.76	0.497
6 h	64.62	75.62	5.01	0.152
12 h	143.70	158.14	7.81	0.220
24 h	185.45	195.19	7.22	0.363
36 h	206.71	211.53	8.18	0.686
48 h	211.81	216.69	7.88	0.671
B (mL/g)	223.48	224.19	8.22	0.890
c (h^{-1})	0.073	0.083	0.005	0.168
Methane content (%)	21.16	22.01	0.93	0.537
Methane production (mL/g)	45.89	49.99	3.73	0.454

[1] NEC: no-ethanol control; CO: ethanol control; [2]SEM: standard error of the mean; B: asymptotic gas production of per gram DM substrate; c: the rate of gas production per hour.

Table S2. Effects of the ethanol carrier on fermentation parameters and DM degradation rate *in vitro*

Items	NEC[1]	CO	SEM[2]	*P*-value
DM degradation rate (%)	62.05	61.57	0.49	0.529
pH	6.92	6.90	0.01	0.495
TVFA (mM)	140.48	143.11	2.85	0.528
Acetate (mM)	75.57	76.79	1.47	0.569
Propionate (mM)	26.89	27.27	0.51	0.611
Butyrate (mM)	20.90	21.44	0.60	0.533
Isobutyrate (mM)	3.09	3.18	0.05	0.198
Valerate (mM)	4.69	4.80	0.09	0.381
Isovalerate (mM)	9.35	9.62	0.18	0.322
mol/100 mol VFA				
Acetate	53.80	53.66	0.08	0.275
Propionate	19.15	19.06	0.08	0.407
Butyrate	14.85	14.98	0.12	0.476
Isobutyrate	2.20	2.22	0.02	0.552
Valerate	3.34	3.35	0.01	0.404
Isovalerate	6.66	6.72	0.07	0.556
Acetate:Propionate	2.81	2.82	0.01	0.735
NH_3-N (mg/dL)	30.62	30.49	0.40	0.826
MCP (mg/dL)	3.07	2.93	0.16	0.536

[1]NEC: no-ethanol control; CO: ethanol control; [2]SEM: standard error of the mean; TVFA: total volatile fatty acid; NH_3-N: ammonia nitrogen; MCP: Microbial Crude Protein.

Table S3. Effects of Lovastatin on gas production, methane production and kinetic parameters in the first *in vitro* fermentation batch

Items	Group[1]				SEM[2]	*P*-value		
	CO	50LV	100LV	150LV		Treatment	Linear	Quadratic
Cumulativegasproduction(mL/g)								
3h	42.13	37.90	37.04	40.36	0.826	0.106	0.378	0.022
6h	88.39[a]	80.71[ab]	78.19[b]	84.84[ab]	1.276	0.014	0.178	0.003
12h	152.83	162.21	149.82	163.87	2.672	0.169	0.371	0.650
24h	194.13	195.18	181.08	197.66	3.122	0.241	0.898	0.214
36h	220.56	214.14	195.89	212.53	3.830	0.123	0.199	0.122
48h	228.83	222.35	201.63	220.75	3.899	0.068	0.168	0.084
B (mL/g)	232.93	227.23	206.09	224.68	4.221	0.121	0.206	0.138
c (h^{-1})	0.08	0.09	0.09	0.09	0.002	0.177	0.043	0.463
Methane content (%)	20.62	19.57	19.98	20.61	0.174	0.075	0.777	0.015
Methane production (mL/g)	48.70[a]	43.03[ab]	40.54[b]	47.08[ab]	1.044	0.012	0.348	0.002

[1]CO: control (without lovastatin); 50LV, 100LV, and 150LV, lovastatin added at 50, 100, and 150 mg/kg DM, respectively; [2]SEM: standard error of the mean; B: asymptotic gas production of per gram DM substrate; c: the rate of gas production per hour. Different superscript letters indicate significant differences ($P < 0.05$).

Table S4. Effects of Lovastatin on fermentation parameters and DM degradation rate in the first *in vitro* fermentation batch

Items	Group[1]				SEM[2]	*P*-value		
	CO	50LV	100LV	150LV		Treatment	Linear	Quadratic
DM degradation rate (%)	62.52	64.54	65.37	64.99	0.628	0.430	0.180	0.365
pH	6.89	6.88	6.87	6.88	0.011	0.896	0.580	0.645
TVFA concentration (mM)	140.13	141.55	141.57	139.30	0.742	0.665	0.725	0.242
Acetate (mM)	75.41	76.62	75.21	74.90	0.406	0.487	0.430	0.367
Propionate (mM)	26.76[b]	27.14[ab]	28.04[a]	26.76[b]	0.177	0.02	0.504	0.011
Butyrate (mM)	20.82	20.75	20.74	20.53	0.172	0.949	0.598	0.851
Isobutyrate (mM)	3.15[ab]	3.14[ab]	3.35[b]	3.13[a]	0.032	0.031	0.553	0.066
Valerate (mM)	4.71	4.68	4.74	4.75	0.053	0.978	0.756	0.852
Isovalerate (mM)	9.28	9.23	9.50	9.24	0.095	0.742	0.850	0.601
mol/100 mol VFA								
Acetate	53.82[ab]	54.13[a]	53.12[b]	53.78[ab]	0.124	0.019	0.238	0.415
Propionate	19.09[b]	19.17[b]	19.81[a]	19.22[ab]	0.094	0.017	0.162	0.048
Butyrate	14.86	14.65	14.65	14.72	0.071	0.735	0.545	0.362
Isobutyrate	2.25[a]	2.22[a]	2.37[b]	2.25[a]	0.018	0.01	0.292	0.127
Valerate	3.36	3.31	3.35	3.41	0.031	0.752	0.573	0.373
Isovalerate	6.62	6.52	6.71	6.63	0.054	0.676	0.671	0.944
Acetate:Propionate	2.82[a]	2.82[a]	2.68[b]	2.80[a]	0.016	<0.001	0.054	0.018
NH_3-N (mg/dL)	31.17[ab]	30.69[b]	32.94[a]	32.52[ab]	0.294	0.009	0.007	0.952
MCP (mg/dL)	4.74	4.85	5.64	5.13	0.143	0.111	0.118	0.254

[1]CO: control (without lovastatin); 50LV, 100LV, and 150LV, lovastatin added at 50, 100, and 150 mg/kg DM, respectively; [2]SEM: standard error of the mean. TVFA: total volatile fatty acid; NH_3-N: ammonia nitrogen; MCP: Microbial Crude Protein. Different superscript letters indicate significant differences ($P < 0.05$).
